# Hip arthroscopy for the management of osteoid osteoma of the acetabulum: a systematic review of the literature and case report

**DOI:** 10.1186/s12891-015-0779-8

**Published:** 2015-10-24

**Authors:** Yousef A. Marwan, Sarantis Abatzoglou, Ali A. Esmaeel, Saad M. Alqahtani, Saleh A. Alsulaimani, Michael Tanzer, Robert E. Turcotte

**Affiliations:** Department of Orthopaedic Surgery, Al-Razi Orthopaedic Hospital, Kuwait City, Kuwait; Division of Orthopaedic Surgery, Montreal General Hospital, McGill University Health Centre, Montreal, QC Canada; Division of Orthopaedic Surgery, Department of Surgery, Faculty of Medicine, Health Sciences Center, Kuwait University, Kuwait City, Kuwait; Department of Orthopaedic Surgery, University of Dammam, Dammam, Saudi Arabia

**Keywords:** Osteoid osteoma, Hip, Acetabulum, Arthroscopy

## Abstract

**Background:**

Intra-articular osteoid osteoma (OO) causes irreversible joint damage. Its treatment of choice is radiofrequency ablation (RFA); however, some areas of the acetabulum are hard to access. Therefore, hip arthroscopy was used to treat this tumor. We aim to systematically review the literature with regards to arthroscopic management of acetabular OO, and to report a further case in which hip arthroscopy was used for treatment.

**Methods:**

PubMed and EMBASE were searched for articles relevant to the arthroscopic management of acetabular OO on December 2, 2014. All articles published on and before that date were reviewed, and studies which met our pre-determined inclusion criteria were included. Articles screening and data abstraction were done by two reviewers independently. We also presented a 31-year-old man with acetabular OO who underwent hip arthroscopy for the management of his tumor after failing to respond to medications and computed tomography scan (CT)-guided RFA.

**Results:**

The initial search revealed 14 studies, of which ten met our inclusion criteria. A total of ten patients underwent hip arthroscopy for the management of acetabular OO. Only two patients were females, and the patients' age ranged from 7 to 47 years. Two patients underwent arthroscopic guided-RFA of the lesion, while the rest underwent excision. The follow-up period ranged from 6 months to 2 years. Success rate was 100 %, and no recurrence was reported. Minor complications (transient impotence and perineal numbness) developed in one patient (10 %). Arthroscopic-guided RFA failed to eliminate the tumor in our additional case. A second trial of CT-guided RFA was successful in treating the patient's condition.

**Conclusions:**

Hip arthroscopy is an effective and safe option for the management of acetabular OO, with success rate exceeding 90 %. Studies of higher level of evidence are required.

**Electronic supplementary material:**

The online version of this article (doi:10.1186/s12891-015-0779-8) contains supplementary material, which is available to authorized users.

## Background

Osteoid osteoma (OO) is a solitary benign osteoblastic tumor, accounting for approximately 11-14 % of all benign bone tumors [1]. This tumor is most commonly located in long bones of the lower extremities of children and young adults [1, 2]. Patients with OO usually present with increasing pain, often nocturnal, that is classically relieved with the use of non-steroidal anti-inflammatory drugs (NSAIDs) [1–5]. Imaging of the tumor using plain radiographs or computed tomography (CT) scans using thin sections reveals a small lytic area that is surrounded by reactive bone sclerosis [1, 6–8]. A nidus, which is a small area of calcification in the center of the lytic lesion, is usually seen with CT scans. When the tumor is located adjacent to the joints or in cancellous bone, significant bone sclerosis may be absent. Magnetic resonance imaging (MRI) might not show the lesion clearly because of the significant edema that surrounds the tumor [8–10]. Moreover, nuclear medicine tests can be useful in diagnosing OO in some cases due to their very high sensitivity [11, 12].

Intra-articular involvement of OO was observed in 10 % of the patients [1, 3]. When the articular surface is involved, the diagnosis becomes more difficult because of the atypical symptoms of joint swelling and limited range of motion of the affected joint [3–5]. Such presentation can be misinterpreted as inflammatory or septic arthritis, synovitis, fracture or osteonecrosis. Disorders such as Legg-Calve-Perthes disease or slipped capital femoral epiphysis can have similar presentation to intra-articular OO in children. Irreversible joint damage can result from the long-standing inflammation and synovitis induced by the tumor [1, 5]. In addition, any intervention to treat an intra-articular OO can result in irreversible joint damage or growth plate injury.

Osteoid osteoma may spontaneously resolve after many years, or following prolonged use of NSAIDs [13, 14]. Other treatment options include radiofrequency ablation (RFA), ultrasound ablation, percutaneous drilling, cryoablation, laser thermocoagulation, arthroscopic resection and open surgery. Nevertheless, not every treatment modality is suitable for intra-articular OO [1, 15–21].

Osteoid osteoma involves the acetabulum in around 0.5 % of the cases [1, 4]. Treating lesions in such location is difficult using CT-guided RFA or open surgery. Such procedures can result in severe cartilage damage; therefore, hip arthroscopy was used in the treatment of acetabular OO [1, 20, 21]. In this study, we aim to systematically review the literature with regards to the use of hip arthroscopy in the management of acetabular OO. We also aim to describe the diagnosis and management of a case of intra-articular OO involving the acetabulum. We hypothesize that hip arthroscopy can be helpful in treating cases of acetabular OO.

## Methods

Two reviewers searched two online databases independently, PubMed and EMBASE, for papers related to arthroscopic treatment of acetabular OO published on or before December 2, 2014. The following subject headings and their related key terms were used: "osteoid osteoma", acetabulum", and "arthroscopy" (see Additional file 1: Appendix 1 for outline of the search strategy). Our inclusion criteria were: (1) all level of evidence, (2) male and female patients, (3) patients of all ages, (4) human studies, (5) English language of publication, (6) OO involving the acetabulum, and (7) arthroscopic treatment. On the other hand, exclusion criteria included: (1) articles published in abstract form only, (2) any non-surgical treatment studies (e.g. cadaveric studies, conservative treatment, review articles …etc.), and (3) patients with non-related diagnosis.

The two reviewers independently screened the titles and abstracts of the retrieved studies. An article was included if one of the reviewers believed it should proceed to full-text review. After that, the two reviewers independently abstracted and recorded relevant data from the included articles in Microsoft Excel 2013 (Microsoft, Redmond, WA). This included demographic information (i.e. authors, year of publication, sample size, study design and level of evidence), clinical data (i.e. age, sex, affected hip and location of the mass), length of follow-up, patients lost to follow-up, arthroscopic technique used (position during surgery, portals, method of removing the mass …etc.), treatment success, recurrence of the mass and complications. Success rate was considered as the primary outcome for this review. Due to the heterogeneity of the included studies, meta-analysis was not done, and a qualitative assessment is being presented. Individual study quality assessments were not performed as well because the included studies are of the level IV evidence.

In addition to the systematic review, we present a 31 year-old male patient who underwent arthroscopic treatment for OO of the left acetabulum. Written informed consent was obtained from the patient for publication of this case report and any accompanying images. A copy of the written consent is available for review by the editor of this journal.

## Results

### Systematic review

**Fig. 1:**
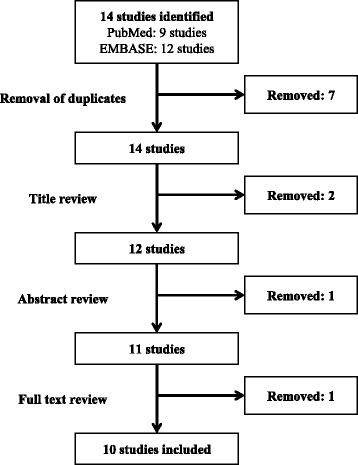
Outline of the systematic search strategy used in this study

The initial search revealed 21 studies. Out of these studies, seven duplicates were removed, and two articles were removed after title review. In addition, one article was excluded after abstract review since there was no available full text; the research was an abstract that was presented in a scientific meeting. Out of the 11 remaining articles, one article was removed after full text review because it described the use of arthroscopy for diagnostic purposes only. Eventually, ten studies were included in our review (Fig. 1). No further studies were found to be eligible for inclusion in our review after screening of the references of the ten included studies.

A meta-analysis was not performed due to the inconsistency of the data in the included studies of this review. All studies were case reports. The two reviewers of this study had no disagreements throughout all stages of the systematic review.

The included studies were conducted in North America, Europe and Asia. Each study reported only one case of acetabular OO that was treated using hip arthroscopy (Table 1) [20–29]. Only two patients were females, and the age of all patients ranged from 7 to 47 years. The left hip was involved in the majority of the patients, and different locations of the mass in the acetabulum were reported. Most patients were operated in the supine position, and underwent hip arthroscopy for excision of the tumor and synovectomy. Two out of the ten patients had arthroscopic guided-RFA of the mass. The follow-up period ranged from six months to 2 years, with one article not reporting the follow-up duration. Success rate was 100 %, and no recurrence of the tumor was noted in any of the patients. In addition, none of the patients required any additional surgical procedure. Complications developed in one patient only in the form of transient post-operative impotence and perineal numbness for 4 weeks.

**Table 1:** Summary of the available literature about the use of hip arthroscopy for the treatment of acetabular osteoid osteoma

Study	Year	Type of study	Sample size	Gender	Age	Affected hip	Location of mass	Position	Portals	Synovectomy	Remarks	Follow-up	Recurrence
Alvarez et al. [23]	2001	Case report	1	M	16	L	Medial wall	NR	Anterolateral; posterolateral; anterior	NR	Excision; post-operative impotence and perineal numbness for 4 weeks	6 months	No
Aşık et al. [29]	2014	Case report	1	M	7	R	Inferior midline of the Y cartilage	Supine	Anterolateral; other portal NR	Yes	RFA and excision	8 months	No
Barnhard & Raven [26]	2011	Case report	1	M	20	R	Anterior of the fovea	NR	Anterolateral; midanterior; anterior to the anterolateral	NR	Excision	NR	No
Chang et al. [25]	2010	Case report	1	F	29	L	Posteroinferior	NR	Anterolateral; posterolateral; anterior	Yes	Excision	1 year	No
Khapchik et al. [22]	2001	Case report	1	M	34	L	Fossa	Lateral decubitus	Anterolateral; posterolateral; anterior	NR	Excision	18 months	No
Lee et al. [24]	2009	Case report	1	M	10	R	Medial wall	Supine	Anterolateral; posterolateral; anterior	NR	Excision	20 months	No
Nehme et al. [27]	2012	Case report	1	M	29	L	Superior	Supine	Anterior; anterolateral; anteroinferior	Yes	Excision	2 years	No
Ricci et al. [20]	2013	Case report	1	F	47	R	Fossa	Supine	Anterolateral; midanterior	Yes	RFA of the tumor	22 months	No
Shoji et al. [28]	2014	Case report	1	M	12	L	Medial wall	Supine	Anterolateral; midanterior	Yes	Excision	14 months	No
Tokis et al. [21]	2014	Case report	1	M	19	L	Posterior column extending to the fovea	Supine	Anterior; anterolateral; anteroinferior	Yes	Excision; Case of recurrence after CT-guided RFA	1 year	No

* M = Male; F = Female; R = Right; L = Left; NR = Not reported; RFA = Radiofrequency ablation

### Case report

**Fig. 2:**
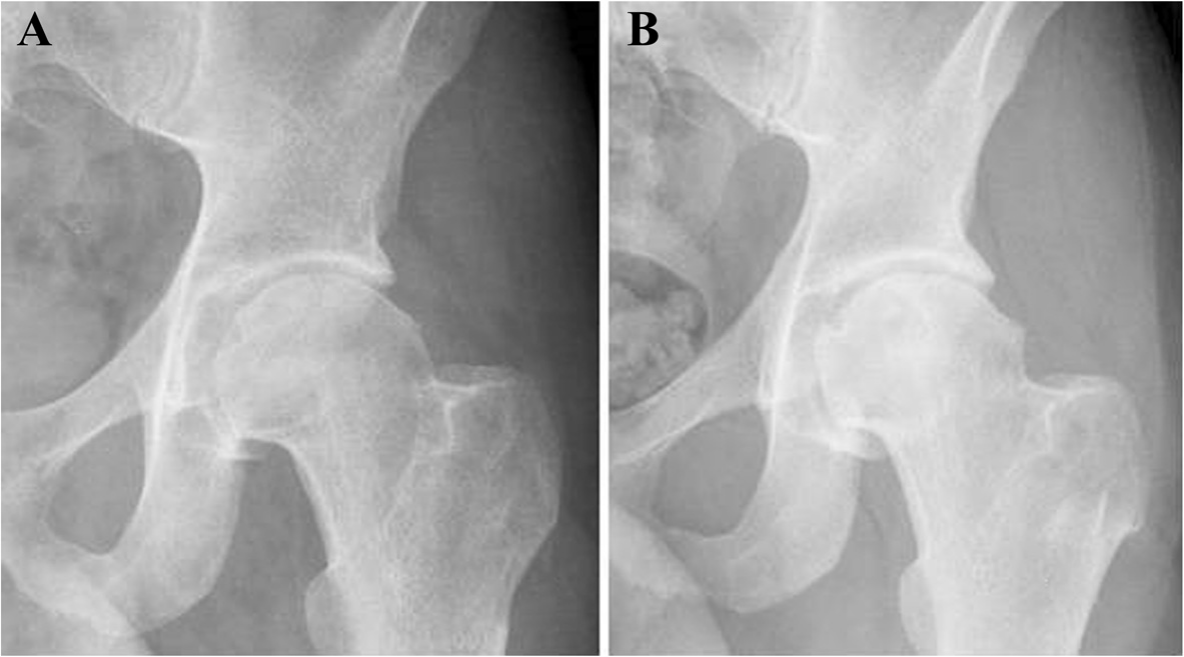
Initial and follow-up radiographs of the left hip of a 31 year-old male with acetabular osteoid osteoma. **a** Plain x-ray of the left hip before undergoing radiofrequency ablation or hip arthroscopy showing mild degenerative changes of the hip joint; **b** Plain x-ray of the left hip of the same patient 44 months following hip arthroscopy and radiofrequency ablation of the acetabular osteoid osteoma not showing further progression of the osteoarthritic changes

**Fig. 3:**
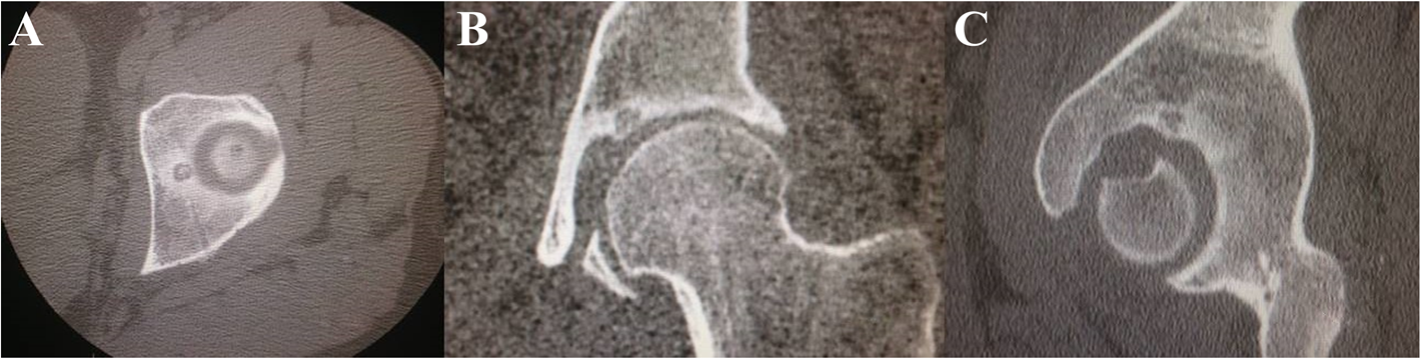
Axial (**a**), coronal (**b**) and sagittal (**c**) views of a computed tomography scan showing a round nidus of osteoid osteoma at the superomedial aspect of the left acetabulum, lying immediately superior and posterior to the fovea in a 31 year-old male with left hip pain

An otherwise healthy 31-year-old male presented with left hip pain of more than one year duration. The pain was mainly located lateral in the iliac area, with minimal pain in the groin, and was radiating to the thigh. The pain was worse at night and waked the patient up from sleep every night. Physical examination of the affected hip revealed normal extension, and limited flexion at 100°, abduction at 30°, adduction at 10°, external rotation at 30° and internal rotation at 15°. The hip was spontaneously adopting an external rotation and abduction position. Plain radiographs suggested osteopenia and joint space thinning and loss of sphericity of the femoral head, but failed to show any tumor (Fig. 2a). The CT scan, MRI and bone scan demonstrated findings suggestive of OO of the left acetabular fossa (Fig. 3).

The patient has tried several NSAIDs before his presentation to our clinic. Nevertheless, all medication trials (including the use of naproxen) failed to relieve his symptoms. Radiofrequency ablation therapy, consisting of three cycles for two minutes at 90 °C with an interval of one minute rest time between each cycle, was performed under CT guidance to treat the lesion. Following the procedure, the patient started to complain of severe pain that was limiting his ability to walk and stand normally even with continuation of NSAIDs. The OO appeared to be unchanged on a CT scan of the left hip that was done 4 months following the RFA. With the possible higher rate of complications of open surgery that requires hip dislocation, the patient agreed for an attempt of arthroscopic excision of the tumor. Based on the CT scans, the tumor was located posterior and superior within the acetabular fossa; therefore, hip arthroscopy was performed using the anterior, anterolateral and posterior portals. The region of the tumor was identified with irregularities of the cartilage at the posterior/superior edge of the fovea. Under fluoroscopic and direct guidance, a burr was placed in the identified region of the tumor. This region was burred, and the cavity of the nidus was identified. The burr could not be placed deeper inside the cavity due to its angulation, thus, the radiofrequency chisel was placed in the region and ablation involved the entire cavity. No intra- or post-operative complications were encountered.

Six weeks after surgery, the patient reported no relief of his pain. A new CT scan of his left hip showed that the OO was still there, while the burned area appeared slightly posterior to the lesion. The patient underwent a second attempt of CT guided RFA of the lesion three weeks later. Improvement of his pain and increased range of motion of the hip were recorded two weeks following that. During the last follow-up visit, 44 months after the last procedure, he reported ignorable mild discomfort during activity. The nocturnal pain was gone, and he regained his ability to walk and stand without any pain. His range of motion improved fully with the exception of adduction at 10° and abduction at 30°. His latest follow-up x-rays showed no progression of the mild degenerative joint changes of the left hip which were noted in the first images (Fig. 2b).

## Discussion

This article is the first to present a systematic review about the use of hip arthroscopy for the management of acetabular OO. Regardless of the technique used, hip arthroscopy was successful in all cases, and recurrence rate was 0 %. Minor complications (i.e. transient impotence and perineal numbness) were reported in one (10.0 %) patient only. The use of hip arthroscopy failed to treat the tumor of our additional case, although the surgeon was an expert in performing such procedures. This emphasizes the difficulties the surgeon may face during arthroscopy for tumors in locations that are hard to reach. Although all areas of the hip can be visualized at the time of surgery, not all areas are amenable to arthroscopic resection. Resection of subchondral lesions requires removal of the overlying cartilage; this is not feasible in lesions in the major weight bearing areas of the acetabulum or femoral head. Moreover, even if a lesion is considered amenable to arthroscopic resection, there are instances in which the instruments’ design does not permit the surgeon to adequately address the lesion as described in our additional case. The difficulty in getting to the lesion because of the femoral head, acetabular location and instrumentation curvature resulted in the inability to burr deep into the subchondral bone and prevented a wider resection of the abnormal area. Our patient developed minimal cartilage/joint damage with the treatment provided; however, he got excellent relief of his pain and regained the range of motion of his joint eventually following the second trial of CT guided RFA.

An intra-articular involvement of OO is rare and can result in misdiagnosis [3–5, 8]. Symptoms usually improve and the tumor can resolve spontaneously with NSAIDs; however, the average time to achieve such results is 33 to 36 weeks [13, 14]. Delay in treatment, or response to it, could result in joint damage for intra- or juxta-articular cases of OO.

Open surgical excision of OO has a high success rate that exceeds 88 % [30–33]. In cases of acetabular OO, open methods provide excellent hip joint access and help in excising or resecting large lesions. Nevertheless, the risk of avascular necrosis of the femoral head and femoral neck fracture is associated with the need for hip dislocation using open surgery. Moreover, rehabilitation programs following the intervention are longer in such cases. In our current review of arthroscopic management of acetabular OO, none of these complications of open surgery were reported among the patients.

Computed tomography-guided RFA represents a very successful modality of treatment of OO of the acetabulum resulting in rapid recovery and minimal complications [16, 17]. Success rate with this minimally invasive intervention reached more than 90 % with short- and long-term follow-up [1, 16, 17, 34, 35]. It requires precise targeting which can be difficult when the lesion is deep into the bone. Placement of the tip of the electrode and the duration of the thermal injury can be adjusted into an attempt to minimize injury to the surrounding tissues. Nevertheless, devitalization of the articular cartilage surrounding the tumor and inability to collect satisfactory specimen for pathological examination are disadvantages of this treatment modality [1, 16, 17, 34–36]. Arthroscopic guidance for RFA of acetabular OO has been described in two cases [20, 29]. It was found to be useful in minimizing the risk of damage to the cartilage with the use of percutaneous RFA and avoid bone disruption with the use of arthroscopic burr. Yet, some areas of the acetabulum might be hard to access. This was observed in the additional case we reported in this article.

### Limitations

Although this systematic review presents important data about the management of acetabular OO using hip arthroscopy, it has some limitations. The literature lacked studies of high quality and high level of evidence. Furthermore, a small number of patients were reported. Due to the fact that the acetabulum is a rare location for OO, researchers were not able to perform studies comparing open versus arthroscopic treatment for this tumor; all available studies were case reports. Moreover, inconsistencies of the surgical techniques used by the surgeons exist, and the tumors were present in different locations of the acetabulum among the patients. The duration of the post-operative follow-up of the patients was short; thus, late recurrence and degenerative changes to the joint could not be assessed. In addition, the results of hip arthroscopy could be affected by the surgeon's experience with this procedure. Although the success rate was high among the reported patients in this systematic review, surgeons who are planning to use a similar treatment option should be competent in performing hip arthroscopy [37].

## Conclusions

Hip arthroscopy appears to be an effective and safe option for the management of acetabular OO, with a success rate of more than 90 %. It should be considered when an image guided approach is not possible or likely to cause more joint damage. Nevertheless, location of the lesion in the acetabulum might limit proper resection. Studies of higher level of evidence are required.

## Additional file

Additional file 1:
**Appendix 1.** Search strategy. (DOC 46 kb)

## Abbreviations

OO: Osteoid osteoma
NSAIDs: Non-steroidal anti-inflammatory drugs
CT: Computed tomography
MRI: Magnetic resonance imaging
RFA: Radiofrequency ablation
