# Supplementary material for: Hip arthroscopy for the management of osteoid osteoma of the acetabulum: a systematic review of the literature and case report
Source: BMC Musculoskelet Disord. 2015 Oct 24;16:318. doi: 10.1186/s12891-015-0779-8 (PMC4620000; doi:10.1186/s12891-015-0779-8)
Supplement: Additional file 1: — Appendix 1. Search strategy. (DOC 46 kb) [file 12891_2015_779_MOESM1_ESM.doc]

Additional file 1: Appendix 1. Search strategy.

|  | PubMed | EMBASE |
| --- | --- | --- |
| Search strategy used | 1. "Osteoma, Osteoid"[Mesh] 2. "Osteoid Osteomas" OR "Osteomas, Osteoid" OR "Osteoid Osteoma" 3. 1 OR 2 4. "Acetabulum"[Mesh] 5. "Acetabulums" OR "Cotyloid Cavity" OR "Cavities, Cotyloid" OR "Cavity, Cotyloid" OR "Cotyloid Cavities" OR "Acetabula" OR "Acetabulas" 6. 4 OR 5 7. "Arthroscopy"[MeSH] 8. "Arthroscopies" OR "Arthroscopic Surgical Procedures" OR "Arthroscopic Surgical Procedure" OR "Procedure, Arthroscopic Surgical" OR "Procedures, Arthroscopic Surgical" OR "Surgical Procedure, Arthroscopic" OR "Surgery, Arthroscopic" OR "Surgical Procedures, Arthroscopic" OR "Arthroscopic Surgery" OR "Arthroscopic Surgeries" OR "Surgeries, Arthroscopic" 9. 7 OR 8 10. 3 AND 6 AND 9 | 1. 'osteoid osteoma'/exp 2. 'osteoid osteomas' OR 'osteomas, osteoid' OR 'osteoid osteoma' 3. 1 OR 2 4. 'acetabulum'/exp 5. 'acetabulum' OR 'acetabular' OR 'acetabulums' OR 'cotyloid cavity' OR 'cavities, cotyloid' OR 'cavity, cotyloid' OR 'cotyloid cavities' OR 'acetabula' OR 'acetabulas' 6. 4 OR 5 7. 'arthroscopy'/exp 8. 'arthroscopy' OR 'arthroscopic' OR 'arthroscopies' OR 'arthroscopic surgical procedures' OR 'arthroscopic surgical procedure' OR 'procedure, arthroscopic surgical' OR 'procedures, arthroscopic surgical' OR 'surgical procedure, arthroscopic' OR 'surgery, arthroscopic' OR 'surgical procedures, arthroscopic' OR 'arthroscopic surgery' OR 'arthroscopic surgeries' OR 'surgeries, arthroscopic' 9. 7 OR 8 10. 3 AND 6 AND 9 11. Limit 10 to English language and human studies |
| Articles retrieved | 9 | 12 |
